# Clinical value of multi-slice spiral computed tomography angiography and three-dimensional reconstruction in the diagnosis of double aortic arch

**DOI:** 10.3892/etm.2014.1763

**Published:** 2014-06-06

**Authors:** XIN CHEN, YANJUAN QU, ZHI-YUAN PENG, JINGGUO LU, XIAOJING MA, WENJUAN HU

**Affiliations:** 1 Department of Radiology, Wuhan Asia Heart Hospital, Wuhan, Hubei 430022, P.R. China; 2 Department of Radiology, Zhongnan Hospital of Wuhan University, Wuhan, Hubei 430071, P.R. China; 3 Department of Cardiology, Wuhan Asia Heart Hospital, Wuhan, Hubei 430022, P.R. China

**Keywords:** double aortic arch, computed tomography, X-ray, angiocardiography

## Abstract

The aim of this study was to evaluate the clincal value of multi-slice spiral computed tomography (MSCT) in the diagnosis of double aortic arch (DAA) and analyze the associated angiography and 3D reconstruction imaging to increase the accuracy of DAA diagnosis. Clinical and imaging data from 15 patients diagnosed with DAA by MSCT were summarized and compared with the corresponding surgical observations. The DAA diagnosis by MSCT for the 15 patients was confirmed by the surgical observations. A total of 13 cases were categorized as type I (double arches are open), including nine with a larger right arch, two with a larger left arch and two with balanced arches. Two cases were categorized as type II (one atretic arch), characterized by left atretic arch. Among the 15 patients, ultrasound diagnosis succeeded in nine cases and failed in the rest. A single malformation was identified in only two cases, whereas the rest had single or multiple combination(s) of intracardiac and extracardiac malformations, including seven with different levels of tracheobronchial stenosis. MSCT was demonstrated to precisely diagnose DAA complicated by malformation and tracheobronchial stenosis. MSCT is an essential therapeutic strategy and serves as a primary method in DAA diagnosis.

## Introduction

Double aortic arch (DAA) is the most common form of vascular ring and accounts for 33–73% of vascular ring malformations (1). DAA entirely or partially surrounds the trachea and esophagus and causes compression and symptoms, including wheezing and difficulties in respiration and swallowing. However, unclear clinical features lead to misdiagnosis or even fatal aorta-tracheal fistula due to tracheal cannula (2). Therefore, early accurate diagnosis is critical (3). In the present study, data from 15 patients who were surgically diagnosed with DAA were summarized and compared with the patients’ multi-slice spiral computed tomography (MSCT) results. DAA classification and MSCT characteristic performance were also analyzed to assess the diagnostic value of MSCT and increase its accuracy in DAA diagnosis.

## Subjects and methods

### Clinical data

A total of 15 cases (nine males and six females; aged between seven days and 66 years old, with an average age of 2.5 years) diagnosed with DAA malformation through computed tomography (CT) and confirmed by surgery were recruited from the Wuhan Asia Heart Hospital (Wuhan, China) between May 2005 and March 2013. All patients underwent a chest X-ray and echocardiography concurrently. The pediatric patients exhibited different levels of wheezing, coughing, and shortness of breath or failure of the respiratory system, and nine of them had feeding difficulties. This study was conducted in accordance with the Declaration of Helsinki and with approval from the Ethics Committee of Wuhan Asia Heart Hospital. Written informed consent was obtained from the subjects or their legal guardians.

### Scanning method

A Brilliance CT 64-channel scanner (Koninklijke Philips N.V., Amsterdam, Holland) or Somatom Definition Flash dual-source CT scanner (Siemens AG, Forchheim, Germany) was utilized for the scanning. Patients incapable of coordination fasted for 4–6 h and were then anesthetized (polyphenols: 2.0–2.5 mg/kg) and scanned. Patients capable of respiratory coordination were asked to hold their breath for 15–20 sec after inspiration during the scanning. Electrocardiogram (ECG)-gating and oxygen inhalation were employed, and ECG and saturation of blood oxygen were detected during the scanning. A non-ionic contrast agent (Ultravist; 370 mgI/ml; Bayer Schering Pharma AG, Berlin, Germany) and a dual syringe injector (Medrad Stellant CT Injector system; Medrad, Pittsburgh, PA, USA) were used, and the injection duration was 15–22 sec. The injection dose and flow speed of the Ultravist were as follows: If ≤12 years old, 2.0–2.5 ml/kg and 2.0–2.5 ml/sec; and if >12 years old, 60–80 ml and 3.0–3.8 ml/sec; followed with 4–15 ml saline injection at 0.8–3.0 ml/sec. If ≤12 years old, delayed scanning was employed after the injections, with a delay time of 15–19 sec; and if >12 years old, bolus tracking and its automatic triggering were employed to monitor the enhancement of the descending aorta, and the view of interest was set in the descending aorta on the aortopulmonary window level, with a threshold value of 100–140 HU. The scanning scope ranged from the upper edge of the sixth cervical vertebra to 1 cm below the lower edge of the apex. The Brilliance CT 64-channel scanning parameters were as follows: 80–120 kV, 100–300 mAsec, pitch of 0.2–0.3, X-ray tube rotation time of 0.4 sec/round, 180×180 to 300×300 mm field of view (FOV), matrix size of 512×512, slice thickness of 0.67 mm, reconstruction interval of 0.33 mm, reconstruction phase 40% and 75%. The Somatom Definition Flash dual-source CT scanning parameters were as follows: 80–120 kV, 100–300mAsec, pitch of 0.2–0.5, X-ray tube rotation time of 0.33 sec/round, 180×180 to 300×300 mm FOV, matrix size of 512×512, slice thickness of 0.75 mm, reconstruction interval of 0.40 mm, and the reconstruction phase was automatically determined by a computer to select the best systolic and diastolic periods. Following the scanning, the original data were uploaded to the Philips Extended Brilliance Workspace (Koninklijke Philips N.V.).

### Medical care of patients

All subjects were asked to wear lead eyeglasses, scarves and aprons below the abdomen to reduce the radiation damage to the thyroid, gonads and eyes.

### Results of the diagnosis and imaging diagnostic methods

All diagnoses were provided by at least two experts in cardiovascular imaging following the independent analysis of the CT imaging results and clinical features according to the principle of DAA pathological anatomy. The tracheal stenosis and its location were also assessed. Agreements were obtained and in-depth discussions were held when disagreements occurred. The post analysis included multi-planar reconstruction (MPR), maximum intensity projection (MIP), minimum intensity projection (MinIP) and volume rendering (VR).

## Results

### Comparison of MSCT with ECG and surgical observations

**Figure 1:**
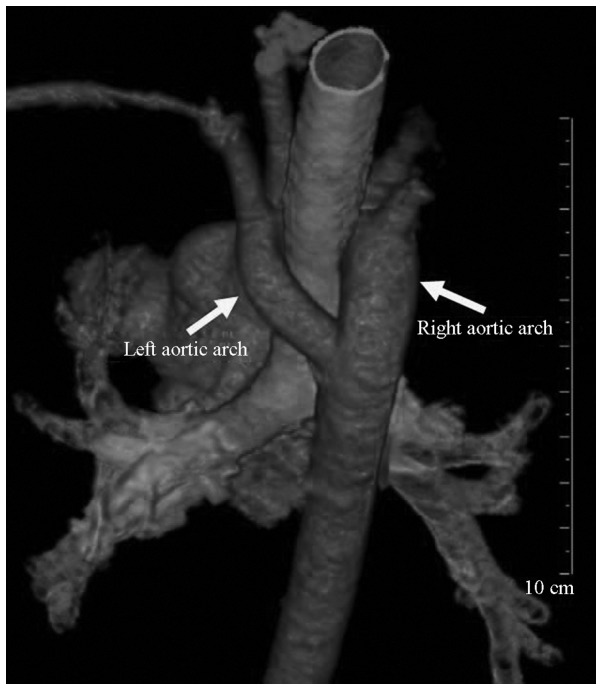
A ten-month-old male infant with DAA. The CT volume-rendered image shows that the aortic arch split into two arches surrounding the trachea (thick right arch and small left arch). DAA, double aortic arch; CT, computed tomography.

**Figure 2:**
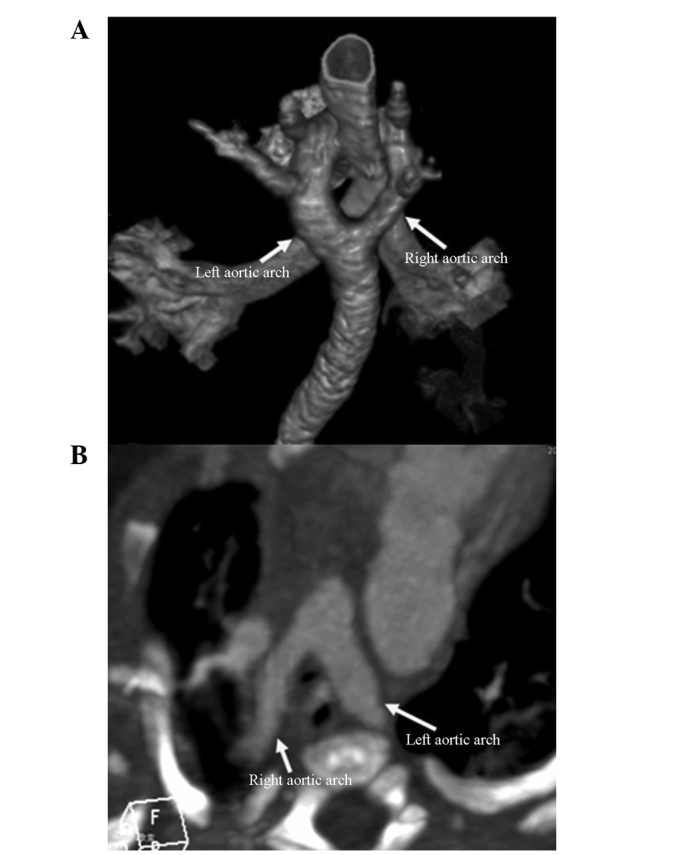
A four-month-old male infant with DAA. (A) The CT volume-rendered image shows that the aortic arch split into two arches surrounding the trachea (thick left arch and small right arch). (B) An axial CT section image. The aortic arch split into two arches surrounding the trachea and esophagus. DAA, double aortic arch; CT, computed tomography.

**Figure 3:**
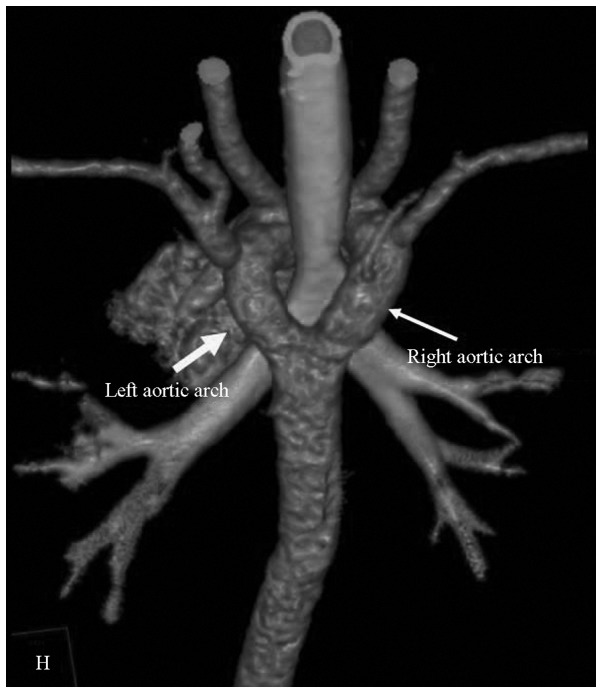
A nineteen-month-old male infant with DAA. The CT volume-rendered image shows that the aortic arch split into two arches surrounding the trachea (similar thickness of two arches). DAA, double aortic arch; CT, computed tomography.

**Figure 4:**
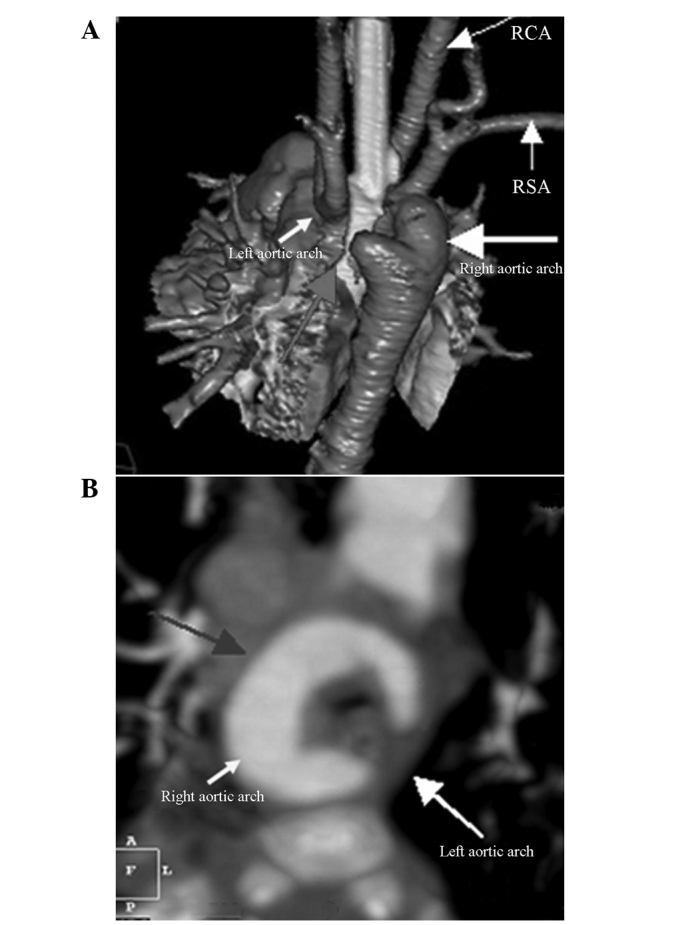
A three-month-old female infant with DAA. (A) The CT volume-rendered image shows that the aortic arch split into two arches surrounding the trachea (right thick arch and left arch atresia). (B) An axial CT section image. The aortic arch split into two arches surrounding the trachea and esophagus (left arch atresia and double arches show ‘C’ shape). DAA, double aortic arch; CT, computed tomography.

All 15 patients with DDA were precisely diagnosed using MSCT and they were as follows: 13 cases of type I (double arches are open), including nine with a larger right arch (Fig. 1), two with a larger left arch (Fig. 2), and two with balanced arches (Fig. 3); and two cases of type II (one atretic arch), which were both cases of left atretic arch (Fig. 4). The ultrasound diagnosis succeeded in nine cases and failed in six. In these six cases, five were diagnosed with right aortic arch, of which three were diagnosed with DAA with a larger right arch and smaller left arch. Two of the three cases had DAA with left atretic arch; one was pre-diagnosed with left aortic arch by ultrasound, which was actually DAA with a larger left arch and a smaller right arch. Among the 15 patients with DAA, only two were identified to have a single malformation, whereas the remainder had single or multiple combination(s) of intracardiac and extracardiac malformations.

### Complication of tracheal stenosis and morphological abnormalities

**Figure 5:**
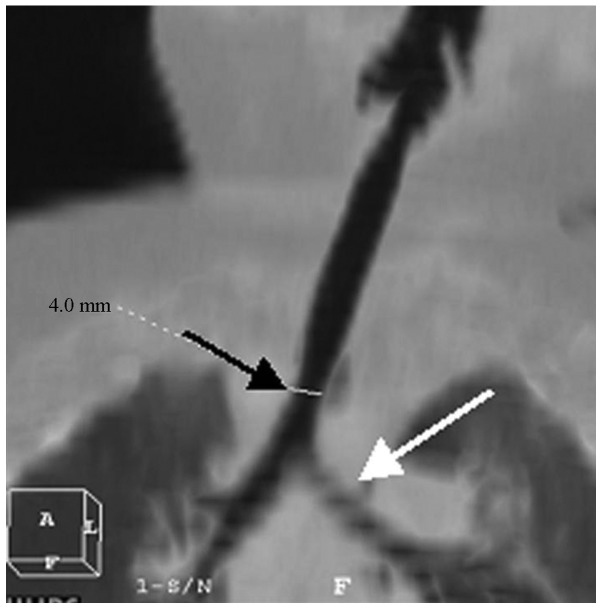
A five-month-old female infant with DAA. The MinIP coronal image shows lower tracheal stenosis (black arrow) and left main bronchial stenosis (white arrow). DAA, double aortic arch; MinIP, minimum intensity projection.

**Figure 6:**
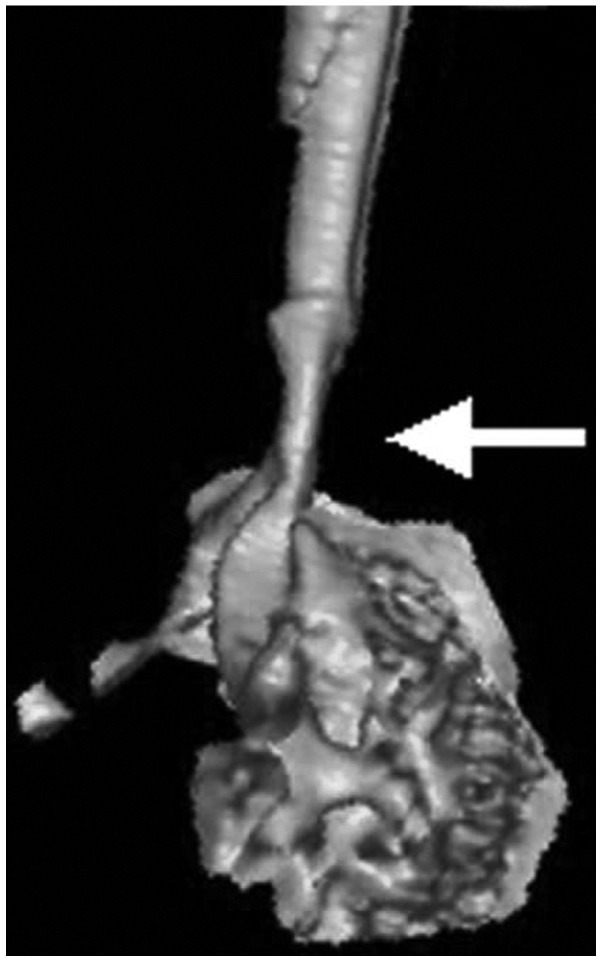
A seven-month-old male infant with DAA. The CT volume-rendered image shows lower tracheal stenosis (white arrow). DAA, double aortic arch; CT, computed tomography.

Seven patients exhibited complications caused by different levels of tracheobronchial stenosis. The MPR, VR and MIP analyses revealed the stenosis of the lower trachea or the bronchus that was involved due to compression (Figs. 5 and 6).

## Discussion

MSCT examination of the DAA anomalies was conducted to clarify the classification and degrees of the combined trachea and bronchial stenosis, as well as the type of the combined endo- and extra-cardiac malformations, which are of clinical significance to the clinicians who select the treatment options. There are few applications of MSCT in cardiovascular disease diagnosis due to the limitations of early CT devices. Therefore, published studies relating to the MSCT diagnosis of DAA anomalies have mainly been case report studies, e.g., that by Choi *et al* (4). With the development of chest imaging tomographic technology, congenital abnormal blood vessel-induced tracheal stenosis is easily and accurately diagnosed and surgically treated, although the risks associated with sedation remain relatively high (5,6). Previous CT equipment prior to 64-slice spiral CT scanners, including single-slice, four-slice and even 16-slice spiral CT, generally takes 10–20 sec to obtain chest images of an infant or toddler. The image motion artifacts are heavy as infants are not able to independently hold their breath. The 64-slice spiral and dual-source CT scanners have a high time resolution rate; and combined with the ECG-gating technique, chest and cardiovascular scanning achieves high-quality images and even avoids motion artifacts. In the present study, the diagnostic accuracy rate of aortic double arch anomalies and types, and tracheal stenosis reached 100%.

DAA is involved in embryonic aortic arch abnormality; the fourth pairs of the bilateral aortic arch and dorsal aortic root fail in degradation and absorption. DAA is pathologically characterized by the normal position of the ascending aorta that bifurcates into the left and right aortic arches in front of the trachea. Pathological classification is into the following two types: i) The two aortic arches are open, generally with a larger right arch and a smaller left arch (7) and ii) one arch is atretic, which has been reported to account for 42–60% of cases and is usually on the left side (8–10). Two of the 15 cases in the present study had DAA with left atretic arch, which is a markedly different frequency compared with that indicated in the literature and is possibly due to the limited number of subjects in the present study. Statistically, DAA is frequently complicated by intracardiac and extracardiac malformations, including tetralogy of Fallot, complete transposition of the great arteries, atrial septal defect, ventricular septal defect and patent ductus arteriosus (11). Among all the cases with aortic arch anomalies in the present study, only one was observed without cardiac malformations. DAA is regarded as the most common type of vascular ring that causes difficulty in respiration due to trachea compression according to a previous study (12). In the present study, seven patients suffered from different degrees of tracheobronchial stenosis.

MSCT is a non-invasive method characterized by a short scanning time, rapid imaging, high time and spatial resolution, and higher isotropic of Z-axis direction. The powerful post-processing function of MSCT allows for the observation of lesions from different angles. MSCT not only clearly reveals the shape and direction of an abnormal vascular ring but also accurately identifies the situation of the tracheal and esophageal compression, which is essential in the diagnosis of pediatric cardiovascular and respiratory diseases (13,14). The MSCT angiography and 3D reconstruction techniques used in the diagnosis of DAA include MIP, VR and MinIP. Axial CT imaging is the basis for DAA diagnosis. The MSCT characteristics of DAA are the visible ‘()’-like vascular structure on the two sides of the trachea in the CT image of the aortic arch in the axial dimension, the narrowing of the trachea, and the enclosed esophagus. If one arch is closed, ‘(’ or ‘)’ are observed in the axial dimension of the aortic arch, and a visible ligament-like structure on the side of the closed aortic arch; similar trachea compression is also observed. It is possible to set the MIP image to an appropriate thickness and orientation to display different pathological changes in the same window. The image is also used to observe cardiac malformations, including atrial and ventricular septal defects, and measure the diameter of the aortic arch. VR is a 3D image that can be rotated at any angle, and intuitively and stereoscopically displays the entire vascular ring and its branches and origin, and the development of the pulmonary arteries, as well as the 3D association between the vascular ring and tracheal compression. VR transparency technology and MinIP show the structural relationship of air-rich trachea, bronchus and lungs with good contrast and sharpness and clearly display anatomical structures with high accuracy and minor human factors (1,15,16).

Angiocardiography is a conventional method that is considered the gold standard in diagnosing DAA due to its high selectivity and excellent capability to indicate anatomy and blood flow in the main pulmonary artery and its branches. However, angiocardiography is invasive, difficult for patients (particularly pediatric patients) to tolerate, and unable to show the compression of the esophagus and trachea as a 2D image. Plain chest radiography and the barium swallow test have been used as auxiliary methods in DAA diagnosis; the two methods reveal indirect signs, including compression of the trachea and esophagus by the aorta and the pulmonary artery and its branches (17). Echocardiography is a non-invasive and simple examination method that identifies the section morphology of the aorta and pulmonary artery and their blood flow in real time. The technique facilitates the diagnosis of complicated intracardiac malformations. However, the disadvantages of echocardiography include the following: i) The display of branches is affected by numerous factors, including the chest bone, pulmonary air, and the technique of the examiner; and ii) poor 3D imaging, which fails to show the vascular spatial orientation and compression of the trachea and esophagus, particularly in patients suffering from double arch deformity with one arch atresia that cannot be accurately diagnosed by echocardiography (18,19). MRI angiography is an effective diagnostic method that does not involve radiation but produces worse image reconstruction, density and time resolution than those of MSCT (20,21). In addition, MRI angiography is not feasible for children who do not cooperate and critically ill patients due to the long duration of MRI scanning (22). The main disadvantage of MSCT angiography is radiation, which is solved by wearing lead eyeglasses, scarves and aprons to minimize the exposure of patients without influencing the diagnosis (23).

In summary, patients with DAA commonly suffer from complications caused by intracardiac and extracardiac malformations and different degrees of airway stenosis, whereas DAA with one-side arch atresia mostly involves left arch atresia. MSCT precisely diagnoses DAA complicated by anomalies and airway stenosis. MSCT is important in treating DAA and thus serves as the primary tool for DAA diagnosis.
